# TGF-β Signaling Interferes With the *Drosophila* Innate Immune and Metabolic Response to Parasitic Nematode Infection

**DOI:** 10.3389/fphys.2019.00716

**Published:** 2019-06-19

**Authors:** Yaprak Ozakman, Ioannis Eleftherianos

**Affiliations:** Infection and Innate Immunity Laboratory, Department of Biological Sciences, The George Washington University, Washington, DC, United States

**Keywords:** *D. melanogaster*, *Heterorhabditis*, immunity, parasitism, TGF-ß signaling

## Abstract

The common fruit fly, *Drosophila melanogaster*, is an outstanding model to study the molecular basis of anti-pathogen immunity. The parasitic nematode *Heterorhabditis gerrardi,* together with its mutualistic bacteria *Photorhabdus asymbiotica,* infects a wide range of insects, including *D. melanogaster*. Recently, we have shown that transforming growth factor-β (TGF-ß) signaling in *D. melanogaster* is regulated in response to parasitic nematode infection. In the current study, we investigated the contribution of two TGF-ß signaling branches, the activin and the bone morphogenetic protein (BMP), to *D. melanogaster* immune function against *H. gerrardi*. We used *D. melanogaster* larvae carrying mutations in the genes coding for the TGF-ß extracellular ligands *daw* and *dpp*. We have demonstrated that the number of circulating hemocytes in uninfected *daw* and *dpp* mutants decreases twofold compared to background controls, yet no significant changes in hemocyte numbers and survival of the TGF-ß mutants are observed upon nematode infection. However, we have shown that nematode-infected *daw* mutants express *Dual oxidase* at higher levels and phenoloxidase activity at lower levels compared to their background controls. To elucidate the contribution of TGF-ß signaling in the metabolic response of *D. melanogaster* to parasitic nematodes, we estimated lipid and carbohydrate levels in *daw* and *dpp* mutant larvae infected with *H. gerrardi*. We have found that both nematode-infected mutants contain lipid droplets of larger size, with *daw* mutant larvae also containing elevated glycogen levels. Overall, our findings indicate that the regulation of activin and BMP branches of TGF-ß signaling can alter the immune and metabolic processes in *D. melanogaster* during response to parasitic nematode infection. Results from this study shed light on the molecular signaling pathways insects activate to regulate mechanisms for fighting potent nematode parasites, which could lead to the identification of novel management strategies for the control of damaging pests.

## Introduction

The fruit fly, *Drosophila melanogaster*, is an established insect model to study innate immune responses against pathogenic infection due to the availability of a wide range of genetic tools (Rämet, 2012). The nematode parasite *Heterorhabditis* forms an excellent experimental tool to dissect the molecular basis of nematode parasitism and mutualism in relation to the insect immune system (Hallem et al., 2007). The nematodes live in mutualistic relationship with the Gram-negative bacteria *Photorhabdus* and together they can infect a variety of insect species (Gerrard et al., 2006; Plichta et al., 2009). *Heterorhabditis* nematodes infect their insect hosts at the infective juvenile stage. Upon entering the insect body cavity, the nematode regurgitates its mutualistic bacteria into the hemolymph to overcome the insect immune response (Stock and Blair, 2008; Castillo et al., 2011).

Investigation of the dynamic interaction between *Heterorhabditis* and *Photorhabdus* species in relation to key aspects of the insect immune system has been facilitated in recent years by the establishment of the tripartite system that involves the fruit fly *D. melanogaster* as the model insect host (ffrench-Constant et al., 2007; Hallem et al., 2007). *D. melanogaster* has evolved certain immune mechanisms to fight against parasitic nematode infection (Castillo et al., 2011). The anti-nematode immunity of *D. melanogaster* includes both humoral and cellular responses in addition to the phenoloxidase cascade that results in melanin formation (Eleftherianos et al., 2016a). Nematode infection also induces stress signaling cascades that result in the synthesis of nitric oxide (NO) and differential regulation of genes involved in the production of reactive oxygen species (ROS) (Castillo et al., 2015; Yadav et al., 2017).

Transforming growth factor-β (TGF-β) signaling pathway is pivotal in cell-cell communication and is involved in several cellular processes, including cell proliferation and differentiation as well as tissue homeostasis and regeneration in mammals (Harradine and Akhurst, 2009). In *D. melanogaster*, it regulates developmental mechanisms including axis formation, body patterning, and morphogenesis (Masucci et al., 1990; Lecuit et al., 1996; Dobens and Raftery, 1998). Similar to vertebrates, the TGF-ß pathway in *D. melanogaster* is composed of two signaling branches: the bone morphogenetic protein (BMP) and the activin branches. The TGF-ß signaling pathway is initiated by the binding of an extracellular ligand to a transmembrane receptor complex of serine/threonine kinases (Raftery and Sutherland, 1999; Shi and Massagué, 2003). BMP signaling includes three ligands: decapentaplegic (dpp), glass-bottom boat (gbb), and screw (scw); and the activin subfamily ligands include *activin-ß* (*actß*), *dawdle* (*daw*), and *myoglianin* (*myo*; Peterson and O’Connor, 2014). Following the activation of the receptor through ligand binding, receptor complex phosphorylates downstream transcription factors that regulate the activation of target genes (Zi et al., 2012).

Recently, a link between TGF-ß signaling pathway activity and interaction with parasitic nematode infection has been found in *D. melanogaster* (Eleftherianos et al., 2016b; Patrnogic et al., 2018a,b). More precisely, both activin and BMP branches of TGF-ß signaling pathway are involved in the immune response to sterile injury and *Micrococcus luteus* bacterial infection in flies (Clark et al., 2011). Also, gene transcript levels of both *dpp* and *daw* are upregulated by *Heterorhabditis gerrardi* and *H. bacteriophora* nematode infection in flies (Eleftherianos et al., 2016b). In addition, inactivation of *dpp* increases fly survival and activates humoral immunity in response to *H. bacteriophora* assault (Patrnogic et al., 2018a).

In the current study, we investigated the potential contribution of activin and BMP branches of TGF-β signaling in *D. melanogaster* immunity against *H. gerrardi* infection. For this, we infected larvae carrying loss-of-function mutations in *daw* or *dpp* with *H. gerrardi* infective juveniles to estimate their survival ability, cellular immune activity including changes in hemocyte numbers, ROS and NO activation, and melanization response. In addition, in order to understand whether TGF-ß signaling regulates the *D. melanogaster* metabolic response to nematode parasites, we measured metabolic processes, including lipid and carbohydrate metabolism in *H. gerrardi*-infected larvae with inactivated *daw* or *dpp* genes. Similar studies in insect model hosts are expected to facilitate our understanding of the link between activation of conserved signaling pathways and their components and host immune capacity in response to potent nematode parasites.

## Materials and Methods

### Fly and Nematode Stocks

All flies were reared on instant *D. melanogaster* diet (Formula 4–24 *D. melanogaster* medium) supplemented with yeast (Carolina Biological Supply), maintained at 25°C, and a 12:12-h light:dark photoperiodic cycle. A fly line with spontaneous dpp^s1^ mutation and a line carrying P-bac insertion Pbac{XP}daw^05680^ were used. Line *w^1118^* was used as the background control in all experiments. All lines were obtained from Bloomington Drosophila Stock Center. Validation of mutant lines was performed using quantitative RT-PCR (Supplementary Figure S1). *H. gerrardi* nematodes were amplified in the larvae of the wax moth *Galleria mellonella* using the water trap technique (White, 1927). Nematodes were used 1–4 weeks after collection.

### Larval Infection

Infections of *D. melanogaster* late 2nd instar larvae with nematodes were performed in microtiter 96-well plates containing 100 μl of 1.25% agarose in each well. Infective juveniles were washed and adjusted to the final density of 100 nematodes in 10 μl of sterile distilled water. Nematodes were pipetted into the wells of the microtiter plate and a single larva was transferred to each well. The plate was covered with a Masterclear real-time PCR film (Eppendorf) and holes were pierced for ventilation. Sterile distilled water was used as negative control. Control larvae maintained with water were able to survive, grow normally, and eventually pupate during the course of the experiment. Infected and uninfected larvae were kept at room temperature in the 96-well plate. At 3- and 24-h time point, infected and uninfected larvae were collected and frozen at −80°C or immediately used in experiments. Each infection was performed three times with biological duplicates. For survival experiments, the survival of larvae kept in nematode-free solution or in nematode solution was counted every 12 h for 60 h. Four independent survival experiments were conducted.

### RNA Analysis

Total RNA was extracted from 5 to 10 *D. melanogaster* larvae, using TRIzol™ reagent according to manufacturer’s protocol. Reverse transcription was performed using the High Capacity cDNA Reverse Transcription Kit (Applied Biosystems) and 350 ng RNA. Quantitative RT-PCR (qRT-PCR) experiments were carried out with gene-specific primers (Table 1) and 3.5 ng cDNA, using iQ SYBR Green Supermix (Bio-Rad Laboratories) and a CFX96 Real-Time PCR detection system (Bio-Rad Laboratories), following the manufacturer’s instructions. Each experiment was run in biological duplicates and repeated three times.

**Table 1:** Primers and their sequences used in quantitative RT-PCR experiments.

Gene	Accession no.	Primer (5′-3′)	Sequence	*T*_m_ (°C)
*Daw*	CG16987	Forward Reverse	GGTGGATCAGCAGAAGGACT GCCACTGATCCAGTGTTTGA	57
*Dpp*	CG9885	Forward Reverse	CCTTGGAGCCTCTGTCGAT TGCACTCTGATCTGGGATTTT	57
*PPO1*	CG5779	Forward Reverse	CAACTGGCTTCGTTGAGTGA CGGGCAGTTCCAATACAGTT	60
*PPO2*	CG8193	Forward Reverse	CCCGCCTATACCGAGA CGCACGTAGCCGAAAC	59
*PPO3*	CG2952	Forward Reverse	GGCGAGCTGTTCTACT GAGGATACGCCCTACTG	58
*Nos*	CG6713	Forward Reverse	AACGTTCGACAAATGCGCAA GTTGCTGTGTCTGTGCCTTC	60
*Duox*	CG3131	Forward Reverse	ACGTGTCCACCCAATCGCACGAG AAGCGTGGTGGTCCAGTCAGTCG	60
*RpL32*	CG7939	Forward Reverse	GATGACCATCCGCCCAGCA CGGACCGACAGCTGCTTGGC	60

### Hemolymph Collection and Total Hemocyte Counts

To extract hemolymph from nematode-infected and uninfected *D. melanogaster* mutant and background control larvae, 10 individuals were bled into 30 μl of 2.5× protease inhibitor cocktail (Sigma P2714). Hemolymph samples were loaded on a hemocytometer and total numbers of cells were counted using 40× magnification of a compound microscope (Olympus CX21). Each experiment was repeated three times.

### Phenoloxidase Activity Assay

*D. melanogaster* larvae were infected with *H. gerrardi* nematodes as previously described, and 10 larvae were collected at 24 h post infection. Phenoloxidase activity was measured according to a previously published protocol with slight modifications (Duvic et al., 2002). Hemolymph of each sample was collected, added to a Pierce^®^ Spin Column, and spun at 4°C and 13,000 rpm for 10 min. Protein concentrations were estimated using Pierce™ BCA Protein Assay Kit (Thermo Fisher Scientific). A mix containing 15 μg of protein, 5 mM CaCl_2_, and 2.5× protease inhibitor were added to 160 μl of L-DOPA solution (in phosphate buffer, pH 6.6) in a clear microplate well. Absorbance was measured at 29°C at 492 nm for 60 min. Absorbance of the blank was subtracted from the absorbance of the samples. Each experiment was run in biological duplicates and repeated three times.

### Metabolic Assays

*D. melanogaster* larvae were infected with *H. gerrardi* nematodes as previously described, and five larvae were collected at 24 h post infection. Larvae were washed several times in cold 1 ml 1× PBS and homogenized in either 100 μl of 1× PBS to determine glucose and glycogen levels or 100 μl of cold PBST (1× PBS + 0.05% Tween 20) to measure triglyceride levels, as previously described (Tennessen et al., 2014). Proteins were quantified by Pierce™ BCA Protein Assay Kit (Thermo Fisher Scientific). To determine the amount of triglycerides in infected and uninfected larvae, samples were diluted 1:1 in PBS-Tween and added to 200 μl of the Infinity™ Triglycerides Liquid Stable Reagent (Thermo Fisher Scientific) in a clear microplate well. Covered samples were incubated at 37°C for 30 min, and absorbance was measured at 540 nm. The amount of triglycerides was determined by the glycerol standard curve. To determine the amount of glucose and glycogen, samples were initially diluted 1:3 in PBS and then separated into two sets for further dilutions. The first set of samples was diluted 1:1 in amyloglucosidase stock solution (1.5 μl of amyloglucosidase in 1 ml of PBS, Sigma) and the second set was diluted 1:1 in PBS. Samples (30 μl) were incubated at 37°C for 60 min in a clear microplate well. Hexokinase (Glucose Assay Reagent, Sigma) reagent (100 μl) was added to each well and samples were incubated at room temperature for 15 min. Absorbance was measured at 340 nm, and the amount of glucose was determined by the second set of samples, which were diluted in PBS. The amount of glycogen was calculated by subtracting the absorbance of glucose from the absorbance of first set of samples (samples diluted with amyloglucosidase stock). The amounts of triglycerides, glucose, and glycogen were calculated relative to the amount of proteins in each sample. Each experiment was run in biological duplicates and repeated three times.

### Lipid Droplet Staining

*D. melanogaster* larvae were infected with *H. gerrardi* nematodes as previously described, and 15 larvae were collected at 24 h post infection. Fat body tissues of larvae were dissected and fixed in 4% paraformaldehyde in PBS at room temperature for 30 min. Tissues were washed two times in PBS and then incubated in the dark for 30 min in 0.05% Nile red diluted 1:1,000 in 1 mg/ml of methanol. Tissues were mounted in ProLong™ Diamond AntiFade Mountant with DAPI (Life Technologies). Images were taken by Zeiss LSM 510 confocal microscope. Quantification of lipid droplet size was performed by selecting the area of the five largest lipid droplets per cell from 20 fat body cells. ImageJ software (National Institutes of Health) was used for quantifications. The experiment was repeated three times.

### Statistical Analysis

GraphPad Prism7 was used for data plotting and statistical analyses. Log-rank (Mantel-Cox) test was used for statistical analysis of the survival results. Statistical analyses of all other experiments were performed using unpaired *t*-test.

## Results

### Nematode Infection Does Not Alter the Survival of TGF-ß Mutants

**Figure 1:**
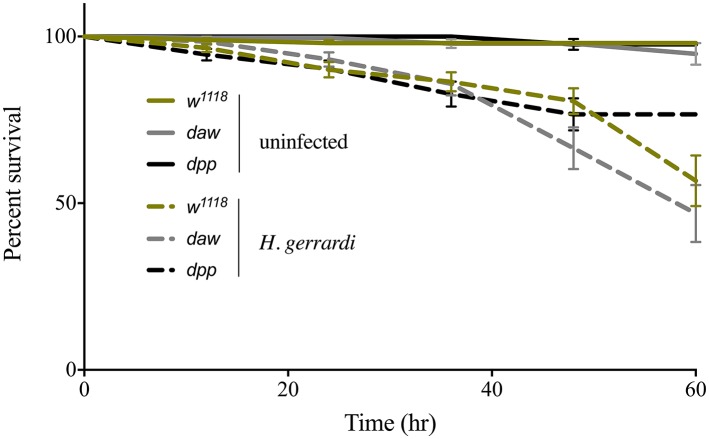
Survival analysis of TGF-ß mutant larvae upon infection with the parasitic nematode *H. gerrardi*. *Daw* and *dpp* mutants together with their background control (*w^1118^*) larvae were infected with *H. gerrardi* symbiotic nematodes. Treatment with water served as negative control. Larval survival was counted every 12 h following infection. There is no significant difference in the survival of uninfected *daw* and *dpp* mutants relative to their background control (*w^1118^*). In addition, no significant difference is found between nematode-infected *daw* or *dpp* mutants and *w^1118^* individuals (non-significant differences are not indicated).

We assessed the ability of *daw* and *dpp* mutant larvae to survive the infection by *H. gerrardi* symbiotic nematodes. For this, we monitored larval survival every 12 h and up to 60 h post nematode infection. We found no significant differences in survival between uninfected TGF-ß mutants and their background control (Figure 1). Also, we did not observe any significant changes in survival between nematode-infected TGF-ß mutants and control individuals. These results indicate that activin and BMP branches of TGF-ß signaling do not contribute to the survival ability of *D. melanogaster* larvae to infection by *H. gerrardi* nematodes.

### Uninfected *daw* and *dpp* Mutants Contain Fewer Circulating Hemocytes

**Figure 2:**
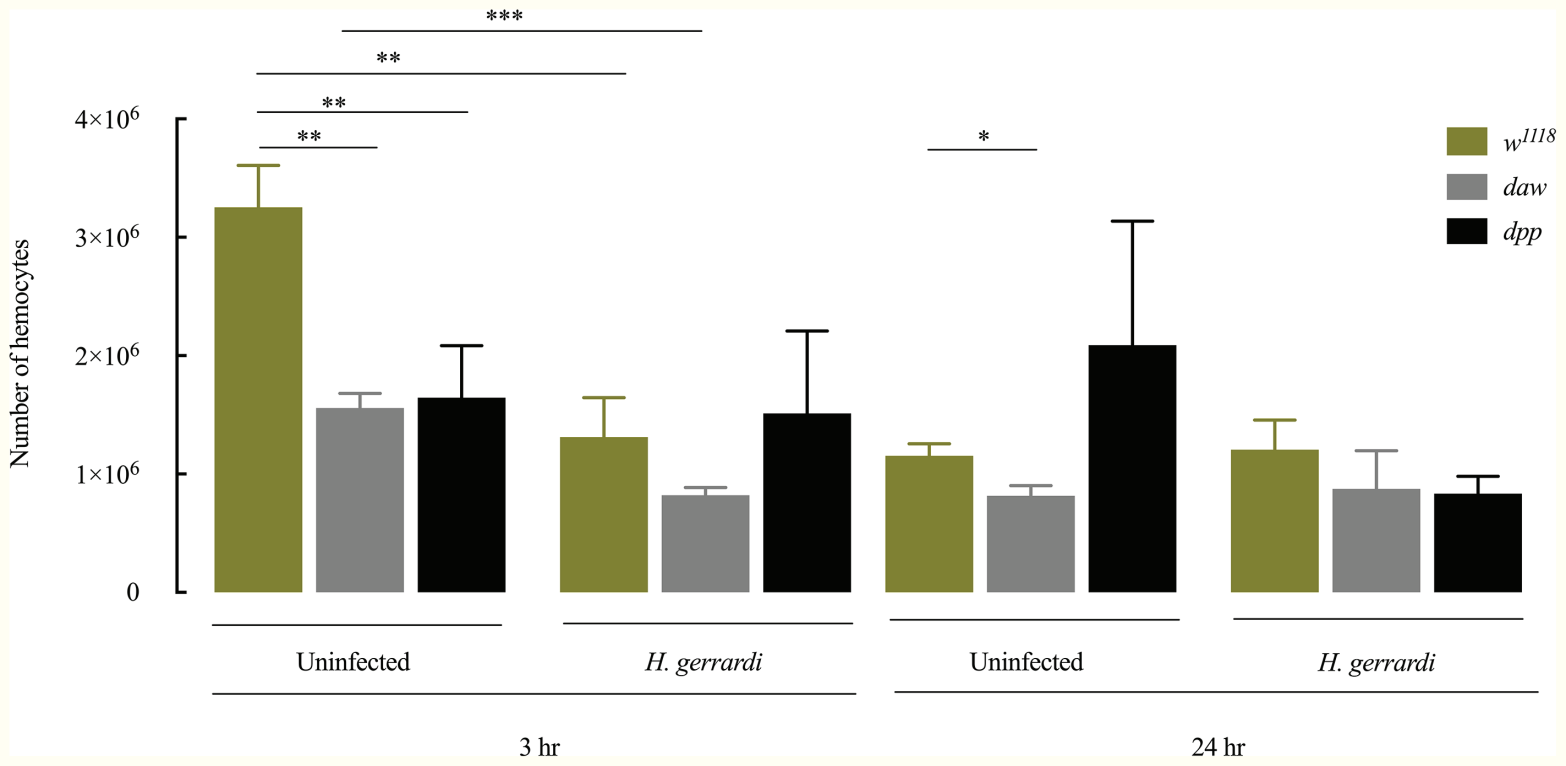
Total number of circulating hemocytes in *Drosophila melanogaster* TGF-ß mutant larvae upon infection with the parasitic nematodes *Heterorhabditis gerrardi*. Hemolymph samples were collected at 3 and 24 h after infection. Numbers of hemocytes in uninfected *daw* and *dpp* mutants are significantly reduced at 3 h relative to their background control (*w^1118^*). *Daw* mutants contain significantly reduced numbers of hemocytes upon nematode infection. Asterisks indicate significant differences between experimental treatments (^*^p < 0.05, ^**^p < 0.01, ^***^p < 0.001; non-significant differences are not indicated).

In *D. melanogaster*, circulating hemocytes play a major role in immune surveillance, and their number can change drastically during pathogenic or non-pathogenic bacterial infection (Eleftherianos et al., 2014; Vlisidou and Wood, 2015; Shokal et al., 2017). To investigate whether inactivating the activin or BMP branches of TGF-ß signaling alters the total number of circulating hemocytes in uninfected *D. melanogaster* or those infected with nematode parasites, we counted hemocytes in larvae carrying loss-of-function mutations in *daw* or *dpp* following treatment with water (control) or infection with *H. gerrardi*. We used two time points to examine changes in hemocyte numbers over time: 3 h post infection as an early time point and 24 h post infection as a later point when nematode infection is established. Both uninfected *daw* and *dpp* mutants contained significantly reduced numbers of hemocytes relative to their *w^1118^* background control at the 3-h time point (*daw*, *p* = 0.0014 and *dpp*, *p* = 0.0078; Figure 2). Similarly, at 24 h, we observed that uninfected *daw* mutants contained significantly fewer hemocytes compared to *w^1118^* larvae (*p* = 0.0119). We then estimated the total number of hemocytes in response to *H. gerrardi* and found that at 3 h post nematode infection, hemocyte numbers in *daw* mutants and *w^1118^* control larvae were significantly lower relative to uninfected controls (*daw*, *p* = 0.0008; control, *p* = 0.0023). However, at the same time point, *dpp* mutants did not show any significant changes in hemocytes numbers in response to *H. gerrardi* infection. We also did not observe any differences in hemocyte numbers between infected or uninfected mutants and *w^1118^* larvae at the 24-h time point. These results indicate that both activin and BMP branches of TGF-ß signaling in *D. melanogaster* are potentially involved in regulating the number of circulating hemocytes in the absence of infection.

### Nematode-Infected *daw* Mutants Express *Duox* at Higher Levels

**Figure 3:**
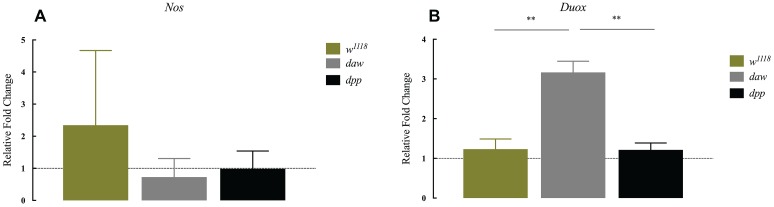
Expression of *Nos* and *Duox* in *Drosophila melanogaster* TGF-ß mutant larvae at 24 h after infection with the parasitic nematodes *Heterorhabditis gerrardi*. *Nos* and *Duox* gene transcript levels in infected larvae are shown as relative fold change normalized to uninfected controls. **(A)** There is no significant change in the expression of *Nos* between the TGF-ß mutants relative to their background control (*w^1118^*). **(B)** Expression of *Duox* in *daw* mutants is upregulated compared to the background controls (^**^p = 0.00419 and ^**^p = 0.0022, respectively; non-significant differences are not indicated).

The production of reactive oxygen species (ROS) and nitric oxide (NO), mediated by dual oxidase (Duox) and nitric oxide synthase (Nos) enzymes, respectively, constitutes an essential regulator of diverse biological processes that include the immune response against bacterial infection (Marletta, 1994; Kuraishi et al., 2013; Eleftherianos et al., 2014). In addition, in mammals in the absence of infection, TGF-ß signaling is potentially regulated by ROS and NO responses (Saura et al., 2005; Jain et al., 2013). However, ROS and NO responses in *D. melanogaster* in the context of parasitic nematode infection and whether TGF-ß signaling participates in the regulation of these processes have not been examined yet. To investigate a potential link between these responses and TGF-ß signaling, we used qRT-PCR and gene-specific primers to determine the transcript levels of (*Nos*) and (*Duox*) in *daw* and *dpp* mutant larvae 24 h after infection with *H. gerrardi* nematodes. We found no statistically significant differences in *Nos* transcript levels between nematode-infected *daw* or *dpp* mutants and their *w^1118^* background controls (Figure 3A). However, the expression of *Duox* in infected *daw* mutants was upregulated compared to *w^1118^* larvae (*p* = 0.00419, Figure 3B) and *dpp* mutants (*p* = 0.0022, Figure 3B). These results suggest a link between the ROS response and the activin branch of TGF-ß signaling in *D. melanogaster* upon response to parasitic nematode infection.

### The Activin Signaling Suppresses the Phenoloxidase Response in Response to *H. gerrardi*

**Figure 4:**
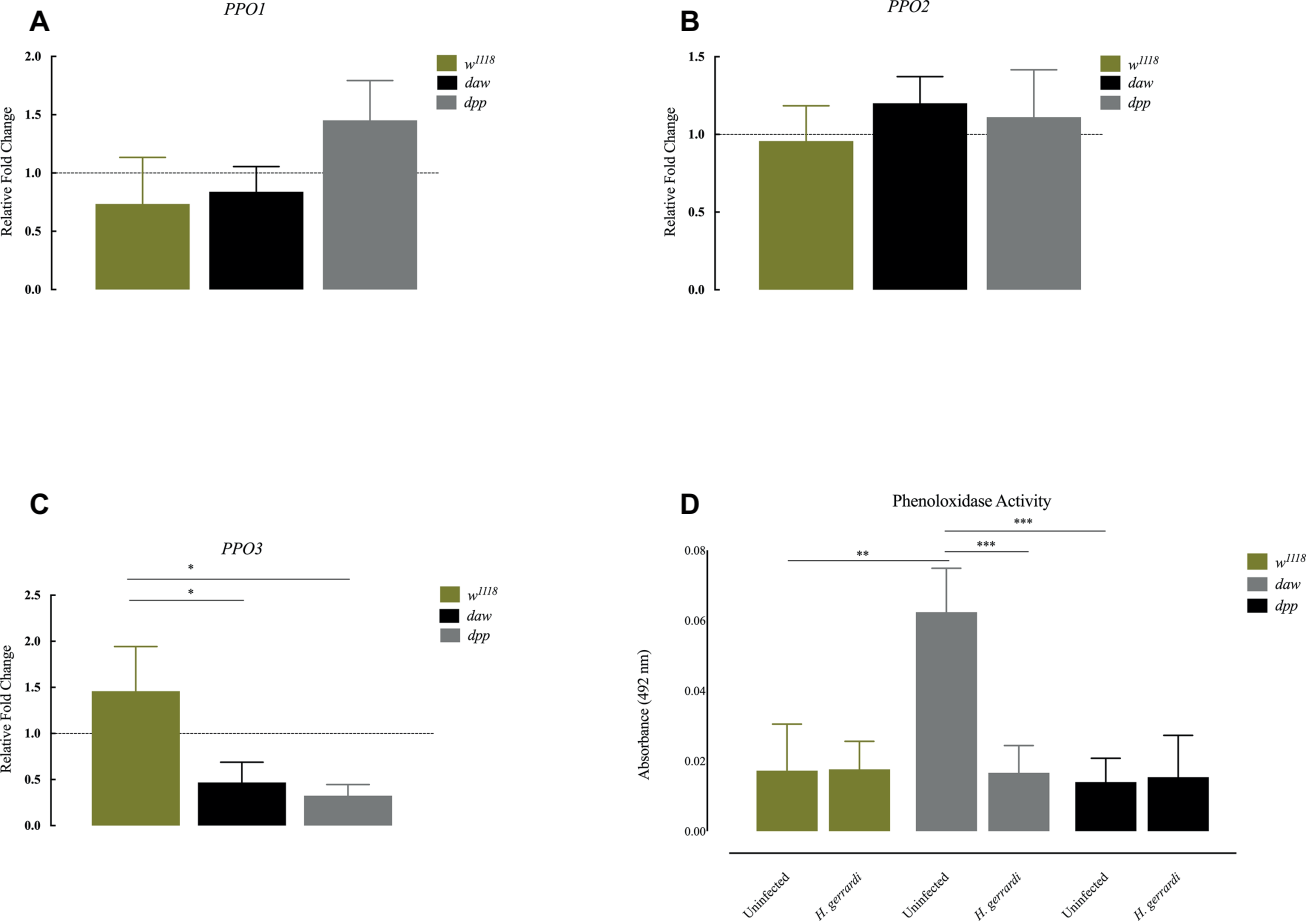
Expression of *PPO* genes and activity of phenoloxidase in *Drosophila melanogaster* TGF-ß mutant larvae at 24 h after infection with the parasitic nematodes *Heterorhabditis gerrardi*. Transcript levels of *PPO1*, *PPO2*, and *PPO3* in nematode-infected larvae are shown as relative fold change normalized to uninfected controls. **(A,B)** There is no significant change in the expression of *PPO1* and *PPO2* between nematode-infected *daw* and *dpp* mutants relative to their background control (*w^1118^*). **(C)** Expression of *PPO3* is reduced in nematode-infected *daw* (*p = 0.0140) and *dpp* mutants (*p = 0.0169) compared to their background control (*w^1118^*). **(D)** Phenoloxidase activity in the hemolymph of *daw* mutants is significantly reduced upon *H. gerrardi* nematode infection compared to uninfected individuals (^**^p = 0.0022, ^***^p = 0.0008; non-significant differences are not indicated).

Previous results indicate that ubiquitous knockdown of *daw* in *D. melanogaster* adult flies results in the formation of melanotic tumors suggesting an association between the TGF-ß activin branch and regulation of the melanization response (Clark et al., 2011). To investigate whether inactivation of TGF-ß signaling in *D. melanogaster* modifies the phenoloxidase response in the context of nematode infection, we challenged BMP and activin loss-of-function mutant larvae with *H. gerrardi* parasites, and 24 h later, we estimated the expression of prophenoloxidase genes *PPO1*, *PPO2*, and *PPO3* using qRT-PCR and gene-specific primers (Tang, 2009). We found no statistically significant differences in the transcript levels of *PPO1* and *PPO2* in *daw* or *dpp* mutants relative to *w^1118^* control larvae upon *H. gerrardi* infection (Figures 4A,B). However, *PPO3* transcript levels were significantly reduced in nematode-infected *daw* (*p* = 0.0140) and *dpp* mutants (*p* = 0.0169) compared to *w^1118^* controls (Figure 4C). We then determined the phenoloxidase enzyme activity in the hemolymph of *daw* and *dpp* mutant larvae infected with *H. gerrardi*. We found that phenoloxidase activity in *daw* mutant larvae was significantly reduced upon nematode infection relative to uninfected counterparts (*p* = 0.0008, Figure 4D). These results imply that the activin branch of TGF-ß signaling in *D. melanogaster* might be involved in suppressing phenoloxidase response in response to *H. gerrardi* nematode infection.

### Size of Lipid Droplets Increases in Nematode-Infected *daw* and *dpp* Mutants

**Figure 5:**
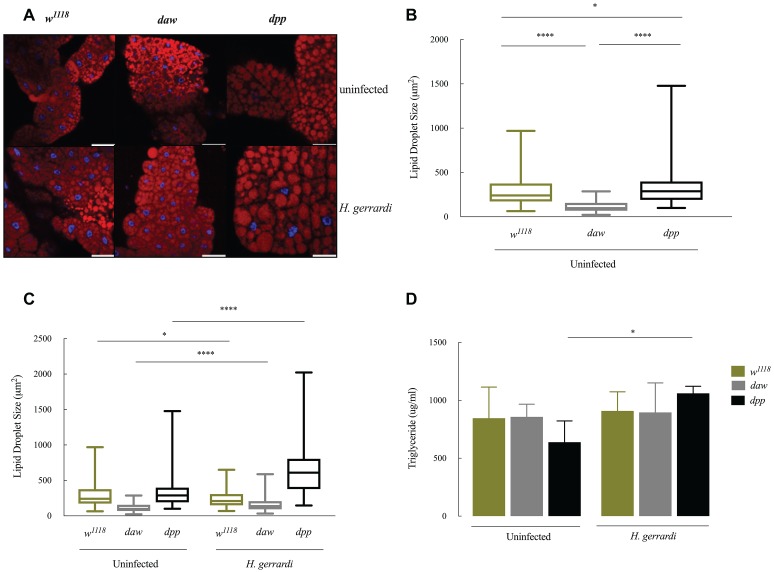
Lipid metabolism in *Drosophila melanogaster* TGF-ß mutant larvae at 24 h upon infection with the parasitic nematodes *Heterorhabditis gerrardi.* **(A)** Confocal microscopy images of lipid droplets in the fat body of *H. gerrardi*-infected and uninfected *D. melanogaster daw* and *dpp* mutants and their background controls (*w^1118^*). **(B)** Size of lipid droplets in the fat body of uninfected *daw* and *dpp* mutants compared to their background controls (*w^1118^*). Size of lipid droplets in uninfected *dpp* mutants significantly increases compared to background controls (*w^1118^*) and *daw* mutants. Size of lipid droplets in uninfected *daw* mutants significantly decreases relative to background controls (*w^1118^*). **(C)** Fat body lipid droplet sizes in *daw* and *dpp* mutants responding to *H. gerrardi* infection relative to uninfected larvae. Size of lipid droplets significantly increases in *daw* and *dpp* mutants infected with *H. gerrardi* nematodes compared to uninfected larvae. Infected background control (*w^1118^*) larvae contain significantly lipid droplets of smaller size relative to uninfected individuals. **(D)** Triglyceride levels in TGF-ß mutant larvae 24 h after infection with *H. gerrardi.* Triglyceride content in nematode-infected *dpp* mutant larvae significantly increases compared to uninfected larvae. Scale bar is 100 μm. Asterisks indicate significant differences between experimental treatments (^*^p < 0.05, ^****^p < 0.0001; non-significant differences are not indicated).

Lipid droplets are vital energy storage organelles found in many organisms. Recent findings suggest that lipid droplets increase in size in *D. melanogaster* infected with *Steinernema carpocapsae* nematodes, which implies a participation in the interaction with certain nematode parasites (Yadav et al., 2018). To determine lipid droplet status in the fat body of TGF-ß-deficient larvae, we stained lipid droplets with Nile red (red) and DAPI (blue) and measured lipid droplet sizes in *daw* and *dpp* loss-of-function mutant larvae (Figures 5A–C). We found that lipid droplets in uninfected *dpp* mutants significantly increased in size compared to *w^1118^* controls (Figure 5B; *p* = 0.0458). However, uninfected *daw* mutants had significantly smaller lipid droplets relative to *w^1118^* larvae (Figure 4B; *p* < 0.0001). Then, we determined lipid droplet sizes 24 h post *H. gerrardi* infection in *daw* and *dpp* mutants. Size of lipid droplets significantly increased in nematode-infected *daw* and *dpp* mutants compared to uninfected larvae (Figure 5C; *p* < 0.0001). Also, nematode infected *w^1118^* controls contained significantly smaller lipid droplets relative to uninfected individuals (Figure 5C; *p* = 0.0221). To further assess changes in lipid metabolism in TGF-ß-deficient *D. melanogaster* larvae in the context of nematode infection, we estimated triglyceride concentrations in *daw* and *dpp* mutant larvae challenged with *H. gerrardi* (Figure 5D). Triglyceride levels in *dpp* mutants infected with the parasitic nematodes were significantly elevated compared to uninfected larvae (*p* = 0.0193), but there were no statistically significant changes in *daw* mutants relative to uninfected individuals. These findings suggest that both BMP and activin branches of TGF-ß signaling in *D. melanogaster* regulate fat body lipid droplet size during response to nematode infection.

### Nematode-Infected *dpp* Mutants Have Elevated Glycogen Levels

**Figure 6:**
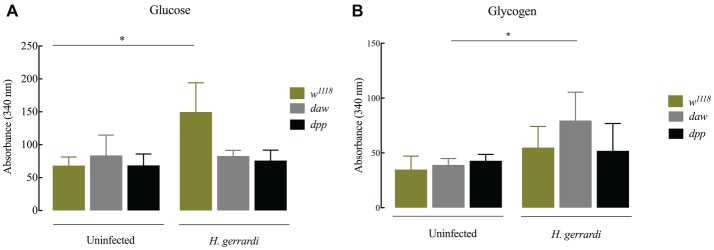
Carbohydrate metabolism in *Drosophila melanogaster* TGF-ß mutant larvae at 24 h upon infection with the parasitic nematodes *H. gerrardi.* **(A)** Level of glucose in infected *daw* and *dpp* mutant larvae. Nematode-infected background controls (*w^1118^*) have significantly higher levels of glucose relative to uninfected larvae (^*^p = 0.0381). **(B)** Level of glycogen in infected *daw* and *dpp* mutant larvae. Nematode-infected *daw* mutants have significantly higher levels of glycogen relative to uninfected larvae (^*^p = 0.0482; non-significant differences are not indicated).

In *D. melanogaster*, glucose is an essential resource for energy production. Glycogen is synthesized and stored several tissues and is required for energy metabolism (Mattila and Hietakangas, 2017). In mammals, there is a direct link between regulation of carbohydrate homeostasis and TGF-ß signaling in the absence of an infection (Yadav et al., 2011). To investigate whether TGF-ß signaling affects carbohydrate metabolism in *D. melanogaster* anti-nematode response, we estimated glucose and glycogen levels 24 h post infection with *H. gerrardi*. We found that upon nematode infection, infected *w^1118^* control larvae had significantly increased glucose levels compared to uninfected individuals (Figure 6A; *p* = 0.0381). However, we did not observe any statistically significant changes in infected *daw* and *dpp* mutant larvae relative to uninfected controls. We found that only nematode-infected *daw* mutants had significantly elevated levels of glycogen relative to uninfected larvae (Figure 6B; *p* = 0.0482). These results indicate that the activin branch of TGF-ß signaling in *D. melanogaster* might participate in modulating glycogen metabolism in the context of nematode infection.

## Discussion

In this study, we explored the contribution of activin and BMP branches of TGF-β signaling in regulating immune activity in *D. melanogaster*. For this, we analyzed changes in larval survival capacity, hemocyte numbers, activation of ROS and NO, and melanization response in uninfected *daw* or *dpp* loss-of-function mutant larvae as well as in larvae infected with *H. gerrardi* parasitic nematodes. We have found a significant decrease in the number of circulating hemocytes in uninfected *daw* and *dpp* mutants compared to their background controls, but no significant change in hemocyte numbers following nematode infection. However, *daw* mutants have higher expression of *Duox* and decreased phenoloxidase activity in response to nematode infection compared to their background controls. We further examined the metabolic activity of *daw* and *dpp* mutant larvae in the presence or absence of *H. gerrardi* infection and found an increase in the size of lipid droplets in both mutants as well as elevated glycogen levels in *daw* mutants upon nematode challenge.

Hemocytes are the central regulators of the cellular immune response against microbial infection in insects, and previous information supports the notion that total number of circulating hemocytes constitutes a robust indication for the level of activation of the cellular arm of the insect innate immune system to act against foreign invaders (Parsons and Foley, 2016). To investigate the contribution of activin and BMP signaling on cellular immune activity in the context of parasitic nematode infection, we estimated changes in hemocyte numbers in uninfected *D. melanogaster daw* and *dpp* mutant larvae and larvae infected with *H. gerrardi* nematodes. Our results indicate that both *daw* and *dpp* uninfected mutants contain significantly fewer hemocytes relative to their background controls. Interestingly, a recent study has shown that the activin branch extracellular ligand Actβ is expressed in sensory neurons, and silencing of Actβ results in fewer hemocyte numbers in *D. melanogaster* larvae in the absence of infection (Makhijani et al., 2017). In agreement with this recent study, our results support the concept that the activin branch of TGF-ß signaling participates in the regulation of hemocyte population at the larval stage of *D. melanogaster*. In contrast, we did not detect any significant changes in hemocyte numbers between nematode-infected *daw* or *dpp* mutants and their background controls, which probably explains the lack of alteration in the survival of the TGF-β mutants in response to *H. gerrardi*. This result further implies that *H. gerrardi* infection has no effect on the total number of circulating hemocytes in *D. melanogaster* larvae. Therefore, current findings suggest that *H. gerrardi* infection does not alter the dynamics of hemocyte numbers in *D. melanogaster* and that the activin and BMP branches of TGF-ß signaling modulate the amount of hemocytes in the uninfected state of the larval stage.

The phenoloxidase enzyme in the melanization cascade regulates the formation of melanin at wound sites and around invading pathogens in the insect hemolymph (Eleftherianos and Revenis, 2011). Previous findings signify that ubiquitous silencing of *daw* in the adult fly causes melanotic tumors mostly in the abdomen, indicating that activin signaling controls the inhibition of the melanization response (Clark et al., 2011). Similarly, here we have found that uninfected *daw* mutants contain significantly higher levels of hemolymph phenoloxidase compared to *dpp* mutants and their background controls. This might suggest a direct or indirect interaction between phenoloxidase activity and activin signaling in response to nematode infection, which will form a subject of our future studies. It is also important to consider that *Photorhabdus* bacteria released from *Heterorhabditis* nematodes into the insect hemolymph secrete molecules, such as rhabduscin and hydroxystilbene, that interfere with the melanization cascade and suppress phenoloxidase activity in the infected insects (Eleftherianos et al., 2007; Crawford et al., 2012). Here, we have found that symbiotic *H. gerrardi* nematodes (containing mutualistic *P. asymbiotica* bacteria) fail to alter phenoloxidase activity in background control and *dpp* mutant larvae, but they are able to suppress the activity of the enzyme in *daw* mutants. This implies that phenoloxidase activity in the hemolymph of *D. melanogaster* larvae during infection with *H. gerrardi* symbiotic nematodes is regulated by the activin signaling of the TGF-β pathway.

Immune cells are required to maintain their cellular metabolism to function efficiently in combating pathogens (Loftus and Finlay, 2016). During infection, *Staphylococcus aureus* induces changes in the host extracellular environment by reducing oxygen and nutrient availability, which generates significant metabolic stress in the mammalian host (Vitko et al., 2015). In the current study, we aimed at understanding the contribution of activin and BMP branches of TGF-ß signaling to metabolic changes in uninfected *D. melanogaster* larvae as well as during nematode infection. It has been previously shown that the BMP ligand *gbb* is essential in the fat body of uninfected *D. melanogaster* larvae to maintain lipid homeostasis and metabolism. *Gbb* loss-of function mutants also display abnormalities in fat body morphology (Ballard et al., 2010). Here, we have also found a significant increase in the size of lipid droplets in uninfected *dpp* mutants compared to background controls indicating the contribution of BMP signaling in maintaining lipid metabolism. However, uninfected *daw* mutants contain significantly smaller lipid droplets suggesting the disruption of lipid metabolism in these larvae. A previous study reported that in *D. melanogaster* embryos histones bound to cytosolic lipid droplets can eliminate both Gram-positive and Gram-negative bacteria *in vitro*. (Anand et al., 2012). In addition, infection with the intracellular bacteria *Mycobacterium tuberculosis*, *M. bovis*, and *M. leprae* leads to the accumulation of lipid droplets in macrophages and Schwann cells in mammalian hosts (D’Avila et al., 2006; Russell et al., 2009; Mattos et al., 2011). Also, infection with the intracellular parasite *Trypanosoma cruzi* in rats induces an increase in the size of lipid droplets in macrophages (Melo et al., 2003). In the fat body of *D. melanogaster,* size of lipid droplets increases in response to infection with the parasitic nematode *S. carpocapsae* carrying the mutualistic bacteria *Xenorhabdus nematophila* (Yadav et al., 2018). In contrast, here we have demonstrated that upon infection with *H. gerrardi* nematodes, which contain the mutualistic bacteria *P. asymbiotica*, size of lipid droplets in the fat body of background control larvae significantly decreases compared to uninfected individuals, suggesting reduced lipid accumulation in this tissue. Such alterations in host lipid metabolism might be an indication of pathogen-specific immune or metabolic responses (Govind, 2008). In our experiments, infection with *H. gerrardi* causes a significant increase in the size of lipid droplets in both *daw* and *dpp* mutant larvae, suggesting that both activin and BMP branches might be involved in the regulation of lipid metabolism in *D. melanogaster* during response to nematode insult.

The current findings highlight the overlapping interactions between the two TGF-ß signaling pathway branches activin and BMP with immune activity and maintenance of lipid and carbohydrate metabolism in uninfected *D. melanogaster* larvae as well as during infection with potent parasitic nematodes. Future research to examine the molecular and functional details of these interactions will contribute toward clarifying the exact role of activin and BMP branches in the host anti-nematode immune response. Due to conservation of innate immune signaling and function in humans, the identification of key immune signaling components will create the basis for identifying novel antihelminth treatment strategies. Alternatively, a better understanding of how parasitic nematodes interact with the immune and metabolic processes of model insects host could potentially lead to the development of innovative tactics for the effective management of agricultural insect pests and vectors of human diseases.

## Author Contributions

YO designed and conducted the experiments, analyzed the data, constructed the figures, interpreted the results, and wrote drafts of the manuscript. IE designed the experiments, interpreted the results, and revised the manuscript.

### Conflict of Interest Statement

The authors declare that the research was conducted in the absence of any commercial or financial relationships that could be construed as a potential conflict of interest.

## References

[ref1] Anand P. Cermelli S. Li Z. Kassan A. Bosch M. Sigua R. (2012). A novel role for lipid droplets in the organismal antibacterial response. elife 1:e00003. 10.7554/eLife.00003, PMID: 23150794 PMC3491588

[ref2] Ballard S. L. Jarolimova J. Wharton K. A. (2010). Gbb/BMP signaling is required to maintain energy homeostasis in *Drosophila*. Dev. Biol. 337, 375–385. 10.1016/j.ydbio.2009.11.011, PMID: 19914231 PMC2838617

[ref3] Castillo J. C. Creasy T. Kumari P. Shetty A. Shokal U. Tallon L. J. (2015). *Drosophila* anti-nematode and antibacterial immune regulators revealed by RNA-Seq. BMC Genomics 16:519. 10.1186/s12864-015-1690-2 26162375 PMC4499211

[ref4] Castillo J. C. Reynolds S. E. Eleftherianos I. (2011). Insect immune responses to nematode parasites. Trends Parasitol. 27, 537–547. 10.1016/j.pt.2011.09.001, PMID: 21982477

[ref5] Clark R. I. Woodcock K. J. Geissmann F. Trouillet C. Dionne M. S. (2011). Multiple TGF-β superfamily signals modulate the adult *Drosophila* immune response. Curr. Biol. 21, 1672–1677. 10.1016/j.cub.2011.08.048, PMID: 21962711 PMC3191266

[ref6] Crawford J. M. Portmann C. Zhang X. Roeffaers M. B. Clardy J. (2012). Small molecule perimeter defense in entomopathogenic bacteria. Proc. Natl. Acad. Sci. USA 109, 10821–10826. 10.1073/pnas.1201160109 22711807 PMC3390839

[ref7] D’Avila H. Melo R. C. Parreira G. G. Werneck-Barroso E. Castro-Faria-Neto H. C. Bozza P. T. (2006). *Mycobacterium bovis* bacillus Calmette-Guérin induces TLR2-mediated formation of lipid bodies: intracellular domains for eicosanoid synthesis in vivo. J. Immunol. 176, 3087–3097. 10.4049/jimmunol.176.5.3087, PMID: 16493068

[ref8] Dobens L. L. Raftery L. A. (1998). *Drosophila* oogenesis: a model system to understand TGF-ß/Dpp directed cell morphogenesis. Ann. N. Y. Acad. Sci. 857, 245–247. 10.1111/j.1749-6632.1998.tb10123.x, PMID: 9917848

[ref9] Duvic B. Hoffmann J. A. Meister M. Royet J. (2002). Notch signaling controls lineage specification during *Drosophila* larval hematopoiesis. Curr. Biol. 12, 1923–1927. 10.1016/S0960-9822(02)01297-6, PMID: 12445385

[ref10] Eleftherianos I. Boundy S. Joyce S. A. Aslam S. Marshall J. W. Cox R. J. (2007). An antibiotic produced by an insect-pathogenic bacterium suppresses host defenses through phenoloxidase inhibition. Proc. Natl. Acad. Sci. USA 104, 2419–2424. 10.1073/pnas.0610525104 17284598 PMC1892976

[ref11] Eleftherianos I. Castillo J. C. Patrnogic J. (2016b). TGF-ß signaling regulates resistance to parasitic nematode infection in *Drosophila melanogaster*. Immunobiology 221, 1362–1368. 10.1016/j.imbio.2016.07.011 27473342 PMC5075508

[ref12] Eleftherianos I. More K. Spivack S. Paulin E. Khojandi A. Shukla S. (2014). Nitric oxide levels regulate the immune response of *Drosophila melanogaster* reference laboratory strains to bacterial infections. Infect. Immun. 82, 4169–4181. 10.1128/IAI.02318-14, PMID: 25047850 PMC4187883

[ref13] Eleftherianos I. Revenis C. (2011). Role and importance of phenoloxidase in insect hemostasis. J. Innate Immun. 3, 28–33. 10.1159/000321931, PMID: 21051882

[ref14] Eleftherianos I. Shokal U. Yadav S. Kenney E. Maldonado T. (2016a). “Insect immunity to entomopathogenic nematodes and their mutualistic bacteria” in The molecular biology of *Photorhabdus* bacteria. ed. ffrench-Constant R. H. (Cham, Switzerland: Springer), 124–148.

[ref15] ffrench-Constant R. H. Eleftherianos I. Reynolds S. E. (2007). Nematode symbionts shed light on invertebrate immunity. Trends Parasitol. 23, 514–517. 10.1016/j.pt.2007.08.021, PMID: 17964855

[ref17] Gerrard J. G. Joyce S. A. Clarke D. J. ffrench-Constant R. H. Nimmo G. R. Looke D. F.. (2006). Nematode symbiont for *Photorhabdus asymbiotica*. Emerg. Infect. Dis. 12, 1562–1564. 10.3201/eid1210.060464, PMID: 17176572 PMC3290952

[ref18] Govind S. (2008). Innate immunity in *Drosophila*: pathogens and pathways. Insect Sci. 15, 29–43. 10.1111/j.1744-7917.2008.00185.x, PMID: 20485470 PMC2871703

[ref19] Hallem E. A. Rengarajan M. Ciche T. A. Sternberg P. W. (2007). Nematodes, bacteria, and flies: a tripartite model for nematode parasitism. Curr. Biol. 17, 898–904. 10.1016/j.cub.2007.04.027, PMID: 17475494

[ref20] Harradine K. A. Akhurst R. J. (2009). Mutations of TGF-ß signaling molecules in human disease. Ann. Med. 6, 403–414. 10.1080/07853890600919911 17008304

[ref21] Jain M. Rivera S. Monclus E. A. Synenki L. Zirk A. Eisenbart J. (2013). Mitochondrial reactive oxygen species regulate transforming growth factor-*β* signaling. J. Biol. Chem. 288, 770–777. 10.1074/jbc.M112.431973, PMID: 23204521 PMC3543026

[ref22] Kuraishi T. Hori A. Kurata S. (2013). Host-microbe interactions in the gut of *Drosophila melanogaster*. Front. Physiol. 4:375. 10.3389/fphys.2013.00375 24381562 PMC3865371

[ref23] Lecuit T. Brook W. J. Ng M. Calleja M. Sun H. Cohen S. M. (1996). Two distinct mechanisms for long-range patterning by decapentaplegic in the *Drosophila* wing. Nature 381, 387–393. 10.1038/381387a0, PMID: 8632795

[ref24] Loftus R. M. Finlay D. K. (2016). Immunometabolism: cellular metabolism turns immune regulator. J. Biol. Chem. 291, 1–10. 10.1074/jbc.R115.693903, PMID: 26534957 PMC4697146

[ref25] Makhijani K. Alexander B. Rao D. Petraki S. Herboso L. Kukar K.. (2017). Regulation of *Drosophila* hematopoietic sites by activin-beta from active sensory neurons. Nat. Commun. 8:15990. 10.1038/ncomms15990, PMID: 28748922 PMC5537569

[ref26] Marletta M. A. (1994). Nitric oxide synthase: aspects concerning structure and catalysis. Cell 78, 927–930. 10.1016/0092-8674(94)90268-2, PMID: 7522970

[ref27] Masucci J. D. Miltenberger R. J. Hoffmann F. M. (1990). Pattern-specific expression of the *Drosophila* decapentaplegic gene in imaginal disks is regulated by 3′ cis-regulatory elements. Genes Dev. 4, 2011–2023. 10.1101/gad.4.11.2011, PMID: 2177439

[ref28] Mattila J. Hietakangas C. (2017). Regulation of carbohydrate energy metabolism in *Drosophila melanogaster*. Genetics 207, 1231–1253. 10.1534/genetics.117.199885, PMID: 29203701 PMC5714444

[ref29] Mattos K. A. Lara F. A. Oliveira V. G. Rodrigues L. S. D’Avila H. Melo R. C. N.. (2011). Modulation of lipid droplets by *Mycobacterium leprae* in Schwann cells: a putative mechanism for host lipid acquisition and bacterial survival in phagosomes. Cell. Microbiol. 13, 259–273. 10.1111/j.1462-5822.2010.01533.x, PMID: 20955239

[ref30] Melo R. C. N. D’Avila H. Fabrino D. L. Almeida P. E. Bozza P. T. (2003). Macrophage lipid body induction by Chagas disease in vivo: putative intracellular domains for eicosanoid formation during infection. Tissue Cell 35, 59–67. 10.1016/S0040-8166(02)00105-2, PMID: 12589730

[ref31] Parsons B. Foley E. (2016). Cellular immune defenses of *Drosophila melanogaster*. Dev. Comp. Immunol. 58, 95–101. 10.1016/j.dci.2015.12.019, PMID: 26748247

[ref32] Patrnogic J. Heryanto C. Eleftherianos I. (2018a). Wounding-induced upregulation of the bone morphogenic protein signalling pathway in *Drosophila* promotes survival against parasitic nematode infection. Gene 673, 112–118. 10.1016/j.gene.2018.06.052 29920363

[ref33] Patrnogic J. Heryanto C. Eleftherianos I. (2018b). Transcriptional up-regulation of the TGF- β intracellular signaling transducer mad of *Drosophila* larvae in response to parasitic nematode infection. Innate Immun. 24, 349–356. 10.1177/1753425918790663 30049242 PMC6830907

[ref34] Peterson A. J. O’Connor M. B. (2014). Strategies for exploring TGF-ß signaling in *Drosophila*. Methods 68, 183–193. 10.1016/j.ymeth.2014.03.016, PMID: 24680699 PMC4057889

[ref35] Plichta K. L. Joyce S. A. Clarke D. Waterfield N. Stock S. P. (2009). *Heterorhabditis gerrardi* n. sp. (Nematoda: Heterorhabditidae): the hidden host of *Photorhabdus asymbiotica* (Enterobacteriaceae: gamma-Proteobacteria). J. Helminthol. 83, 309–230. 10.1017/S0022149X09222942, PMID: 19216823

[ref36] Raftery L. A. Sutherland D. J. (1999). TGF-beta family signal transduction in *Drosophila* development: from mad to Smads. Dev. Biol. 210, 251–268. 10.1006/dbio.1999.9282, PMID: 10357889

[ref37] Rämet M. (2012). The fruit fly *Drosophila melanogaster* unfolds the secrets of innate immunity. Acta Paediatr. 101, 900–905. 10.1111/j.1651-2227.2012.02740.x, PMID: 22606959

[ref39] Russell D. G. Cardona P. J. Kim M. J. Allain S. Altare F. (2009). Foamy macrophages and the progression of the human tuberculosis granuloma. Nat. Immunol. 10, 943–948. 10.1038/ni.1781, PMID: 19692995 PMC2759071

[ref40] Saura M. Zaragoza C. Herranz B. Griera M. Diez-Marques L. Rodriguez-Puyol D.. (2005). Nitric oxide regulates transforming growth factor-beta signaling in endothelial cells. Circ. Res. 97, 1115–1123. 10.1161/01.RES.0000191538.76771.66, PMID: 16239590

[ref41] Shi Y. Massagué J. (2003). Mechanisms of TGF-ß signaling from cell membrane to the nucleus. Cell 113, 685–700. 10.1016/S0092-8674(03)00432-X, PMID: 12809600

[ref42] Shokal U. Kopydlowski H. Eleftherianos I. (2017). The distinct function of *Tep2* and *Tep6* in the immune defense of *Drosophila melanogaster* against the pathogen *Photorhabdus*. Virulence 88, 1668–1682. 10.1080/21505594.2017.1330240 PMC5810505 28498729

[ref43] Stock S. P. Blair H. G. (2008). Entomopathogenic nematodes and their bacterial symbionts: the inside out of a mutualistic association. Symbiosis 46, 65–75. PMID: 21249957

[ref44] Tang H. (2009). Regulation and function of the melanization reaction in *Drosophila*. Fly 3, 105–111. 10.4161/fly.3.1.7747 19164947

[ref45] Tennessen J. M. Barry W. E. Cox J. Thummel C. S. (2014). Methods for studying metabolism in *Drosophila*. Methods 68, 105–115. 10.1016/j.ymeth.2014.02.034, PMID: 24631891 PMC4048761

[ref46] Vitko N. P. Spahich N. A. Richardson A. R. (2015). Glycolytic dependency of high-level nitric oxide resistance and virulence in *Staphylococcus aureus*. MBio 6:e00045-15. 10.1128/mBio.00045-15 25852157 PMC4453550

[ref47] Vlisidou I. Wood W. (2015). *Drosophila* blood cells and their role in immune responses. FEBS J. 282, 1368–1382. 10.1111/febs.13235, PMID: 25688716

[ref48] White G. F. R. (1927). A method for obtaining infective nematode larvae from cultures. Science 66, 302–303. 10.1126/science.66.1709.302-a, PMID: 17749713

[ref49] Yadav S. Daugherty S. Shetty A. C. Eleftherianos I. (2017). RNAseq analysis of the *Drosophila* response to the entomopathogenic nematode *Steinernema*. G3 7, 1955–1967. 10.1534/g3.117.041004, PMID: 28450373 PMC5473771

[ref50] Yadav S. Frazer J. Banga A. Pruitt K. Harsh S. Jaenike J.. (2018). Endosymbiont-based immunity in *Drosophila* melanogaster against parasitic nematode infection. PLoS One 13:e0192183. 10.1371/journal.pone.0192183, PMID: 29466376 PMC5821453

[ref51] Yadav H. Quijano C. Kamaraju A. K. Gavrilova O. Malek R. Chen W.. (2011). Protection from obesity and diabetes by blockade of tgf-beta/smad3 signaling. Cell Metab. 14, 67–79. 10.1016/j.cmet.2011.04.013, PMID: 21723505 PMC3169298

[ref52] Zi Z. Chapnick D. A. Liu X. (2012). Dynamics of TGF-ß/Smad signaling. FEBS Lett. 586, 1921–1928. 10.1016/j.febslet.2012.03.063, PMID: 22710166 PMC4127320

