# Supplementary material for: TGF-β Signaling Interferes With the Drosophila Innate Immune and Metabolic Response to Parasitic Nematode Infection
Source: Front Physiol. 2019 Jun 19;10:716. doi: 10.3389/fphys.2019.00716 (PMC6611403; doi:10.3389/fphys.2019.00716)
Supplement: Supplementary file 1 [file Table_1.DOCX]

**TGF-β signaling interferes with the *Drosophila* innate immune and metabolic response to parasitic nematode infection**

**Yaprak Ozakman, Ioannis Eleftherianos**

**Supplementary Information**


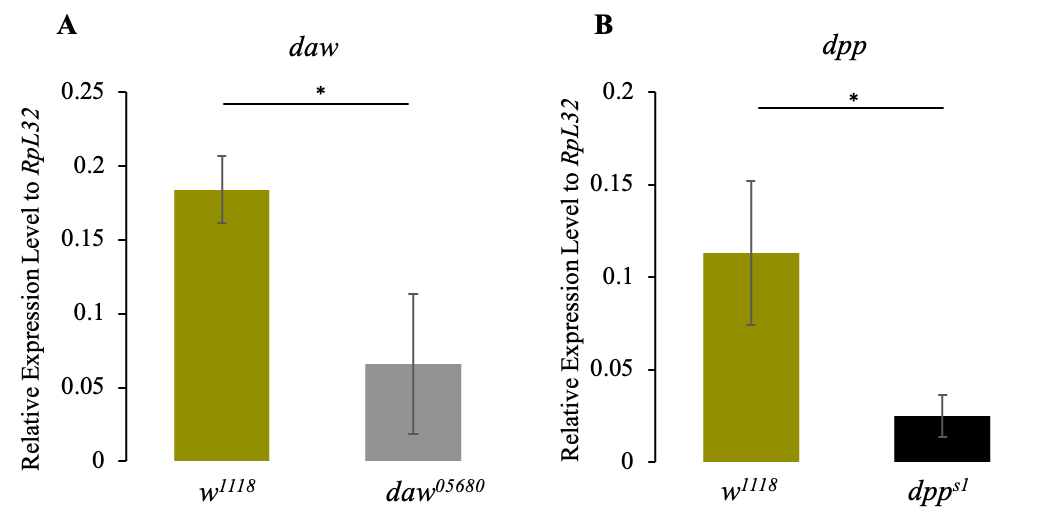


**Figure S1.** Quantitative RT-PCR validation of the *Drosophila melanogaster* *daw* and *dpp* mutant larvae. **(A)** *Daw* mutants have significantly lower expression of *daw* compared to their background control (*w^1118^*) larvae. **(B)** *Dpp* mutants show significantly reduced *dpp* expression relative to their background control (*w^1118^*) larvae (*P < 0.01). Gene expression levels are normalized to *RpL32*.
